# *Porphyromonas gingivalis* LPS Priming Enhances TLR2‐Dependent Chemokine Responses in Human Periodontal Ligament Fibroblasts

**DOI:** 10.1002/cre2.70513

**Published:** 2026-11-04

**Authors:** Masayo Abe, Shintaro Oda, Hiroshi Sakagami, Megumi Inomata

**Affiliations:** ^1^ Department of Oral Biology and Tissue Engineering, Division of Microbiology and Immunology Meikai University School of Dentistry Sakado Japan; ^2^ Research Institute of Odontology (M‐RIO) Meikai University School of Dentistry Sakado Japan

**Keywords:** inflammatory responses, periodontal ligament fibroblasts, TLR2, TLR4, Toll‐like receptors

## Abstract

**Objectives:**

Periodontal ligament fibroblasts (PDLFs) contribute to periodontal inflammation by sensing bacterial components through Toll‐like receptors (TLRs). This study investigated the distinct inflammatory and regulatory responses of PDLFs to TLR2 stimulation and ultrapure *Porphyromonas gingivalis* lipopolysaccharide (*P. g*. LPS), used as a TLR4 ligand, and examined whether prior exposure to *P. g*. LPS modulates subsequent TLR2‐mediated signaling.

**Materials and Methods:**

Human PDLFs were stimulated with the TLR2 ligand Pam3CSK4 or ultrapure *P. g*. LPS. Chemokine gene expression was quantified by real‐time polymerase chain reaction, and IL‐8 and CCL5 concentrations in culture supernatants were measured by enzyme‐linked immunosorbent assay. Activation of the mitogen‐activated protein kinase and nuclear factor‐κB pathways and TLR2 and TLR4 protein expression were examined by western blotting. Cells were pre‐stimulated with *P. g*. LPS and subsequently exposed to Pam3CSK4, either with or without washing to remove the residual stimulus. TLR‐related molecules were also evaluated at the mRNA level.

**Results:**

Pam3CSK4 consistently induced *IL8* and *CCL5* mRNA expression and activated the mitogen‐activated protein kinase and nuclear factor‐κB pathways, whereas *P. g*. LPS alone induced only minimal inflammatory signaling. Pre‐stimulation with *P. g*. LPS enhanced subsequent TLR2‐dependent responses in a manner dependent on stimulus persistence. ELISA analyses showed that IL‐8 and CCL5 secretion patterns were generally consistent with their corresponding mRNA expression profiles. Western blotting confirmed the expression of TLR2 and TLR4 proteins in PDLFs.

**Conclusions:**

These findings indicate that *P. g*. LPS primes PDLFs for enhanced TLR2‐dependent inflammatory responses, particularly under conditions of persistent *P. g*. LPS exposure.

## 1 Introduction

Periodontitis is a chronic inflammatory disease characterized by progressive destruction of the tooth‐supporting tissues and is among the most prevalent infectious inflammatory diseases worldwide (Kinane et al. 2017). The oral cavity harbors a complex microbial community that typically exists in symbiosis with the host. Disruption of this balanced microbial ecosystem can lead to dysbiosis, triggering host immune responses, and promoting chronic inflammation and tissue destruction (Hajishengallis and Lamont 2021).

Innate immune recognition of microbial components in periodontal tissues is largely mediated by pattern‐recognition receptors, particularly Toll‐like receptors (TLRs) (Mahanonda and Pichyangkul 2007). Among these, TLR2 and TLR4 are essential for sensing bacterial components derived from periodontal pathogens. TLR2 primarily recognizes bacterial lipoproteins and lipopeptides, whereas TLR4 detects lipopolysaccharide (LPS) (Hajishengallis and Lamont 2021). Activation of these receptors initiates MyD88‐dependent signaling pathways leading to the activation of nuclear factor‐κB (NF‐κB) and mitogen‐activated protein kinases (MAPK) and subsequently resulting in the production of inflammatory cytokines and chemokines (Kawasaki and Kawai 2014). In addition, TLR4 activates the TRIF‐dependent pathway, which induces type I interferon responses such as IFNB1 and upregulates negative feedback regulators including tumor necrosis factor alpha‐induced protein 3 (TNFAIP3) and nuclear factor kappa‐B inhibitor alpha (NFKBIA), both of which function to restrain excessive NF‐κB–mediated inflammation (Kawasaki and Kawai 2014).

Periodontal ligament fibroblasts (PDLFs) are predominant stromal cells in the periodontal ligament that are essential for maintaining periodontal tissue homeostasis. In addition to their structural functions, accumulating evidence suggests that fibroblasts participate actively in the innate immune response by sensing bacterial components and producing inflammatory mediators (Bartold et al. 2000). PDLFs express functional TLRs and respond to bacterial stimuli, such as LPS, by producing cytokines and chemokines involved in periodontal inflammation (Zhang and Li 2015). However, compared with professional immune cells, the regulatory mechanisms governing TLR signaling and integrating signals from different TLR pathways in fibroblasts remain incompletely understood.

Recent single‐cell transcriptomic studies have revealed that fibroblasts in periodontal tissues are not a uniform population, but include distinct inflammatory subsets that contribute to immune cell recruitment and disease progression (Mo et al. 2024). These findings support the concept that fibroblasts actively regulate periodontal inflammation rather than solely serving as structural cells, highlighting the importance of understanding how fibroblasts integrate microbial signals.

In the periodontal environment, host cells are exposed to multiple bacterial components, either simultaneously or sequentially. Therefore, interactions between distinct TLR signaling pathways may critically influence the magnitude and quality of inflammatory responses. In immune cells, prior stimulation with TLR4 can induce endotoxin tolerance, a phenomenon characterized by reduced responsiveness to subsequent stimulation (Biswas and Lopez‐Collazo 2009). Crosstalk between TLR signaling pathways modulates downstream inflammatory responses and shapes host immunity (Biswas and Lopez‐Collazo 2009). Negative‐regulatory molecules such as tumor necrosis factor alpha‐induced protein 3 (TNFAIP3) and nuclear factor kappa‐B inhibitor alpha (NFKBIA) are crucial for controlling TLR‐mediated inflammatory signaling; however, how these regulatory mechanisms function in PDLFs remains unclear. Furthermore, the influence of stimulus persistence—such as continuous versus transient exposure to bacterial components—on inflammatory responses in fibroblasts has not been fully elucidated.

This study aimed to characterize the responses of human PDLFs to Pam3CSK4 and ultrapure *Porphyromonas gingivalis* lipopolysaccharide (*P. g*. LPS) and to determine how prior exposure to *P. g*. LPS modulates subsequent TLR2‐mediated inflammatory responses. By incorporating experimental conditions with and without washing after *P. g*. LPS stimulation, we evaluated how the persistence of *P. g*. LPS exposure affected inflammatory outcomes.

## 2 Materials and Methods

### 2.1 Reagents

Ultrapure *P. g*. LPS and the synthetic triacylated lipopeptide Pam3CSK4 were purchased from InvivoGen (San Diego, CA, USA). Phosphorylation‐specific antibodies against p38 (Cat. No. 4631), ERK (Cat. No. 4376), and IRF3 (Cat. No. 29047), and antibodies against total p38 (Cat. No. 9212), total ERK (Cat. No. 9102), IκBα (Cat. No. 9242), TLR2 (Cat. No. 12276), and TLR4 (Cat. No. 38519) were obtained from Cell Signaling Technology (Danvers, MA, USA). Anti‐α‐tubulin pAb HRP‐DirecT (Cat. No. PM054‐7) was purchased from Medical & Biological Laboratories Co. Ltd. (MBL; Nagoya, Japan).

### 2.2 Cell Culture

Primary human PDLFs were purchased from ScienCell (Catalog No. 2630; ScienCell Research Laboratories, Tokyo, Japan). According to the manufacturer, donor characteristics such as sex and age, as well as lot numbers, cannot be specified when ordering this product. Cells were cultured in Dulbecco's modified Eagle's medium (DMEM; FUJIFILM Wako Pure Chemical Corporation, Osaka, Japan) supplemented with 10% heat‐inactivated fetal bovine serum (FBS; Thermo Fisher Scientific, Waltham, MA, USA) and 1% penicillin‐streptomycin. Cells were maintained in 10‐cm culture dishes at 37°C in a humidified incubator with 5% CO_2_ as described previously (Abe et al. 2026). Cells at passages 5–7 were used for all experiments. In selected experiments, primary PDLFs isolated from human periodontal ligament tissue were used to confirm reproducibility. Isolation was performed as described previously (Kantoh et al. 2010), and the cells were maintained under the same culture conditions. These PDLFs were obtained from periodontal ligament tissue collected from a mandibular first premolar extracted from a 12‐year‐old female for orthodontic reasons. The use of donor‐derived PDLFs was approved by the Institutional Review Board of the Meikai University Ethics Committee (approval number A0808), and written informed consent was obtained before tissue collection.

### 2.3 RNA Isolation and Quantitative Reverse‐Transcription PCR (qRT‐PCR)

Total RNA was extracted using the NucleoSpin RNA kit (TaKaRa Bio, Shiga, Japan). A total of 500 ng RNA was reverse‐transcribed using ReverTra Ace reverse transcriptase (Toyobo, Osaka, Japan) with oligo(dT)_18_ and random hexamer primers. qRT‐PCR was performed using TB Green Premix Ex Taq II (Tli RNase H Plus; TaKaRa Bio) on a LightCycler 480 system (Roche Diagnostics, Basel, Switzerland). Predesigned primer sets were purchased from TaKaRa Bio; therefore, primer sequences are not shown. PCR conditions were as follows: 95°C for 30 s, followed by 40 cycles of 95°C for 5 s and 60°C for 20 s. Gene expression levels were calculated using the ΔΔCt method and normalized against ACTB expression.

### 2.4 Immunoblotting

Cells seeded in six‐well plates were lysed with 250 µL of lysis buffer (Inomata et al. 2022; Inomata et al. 2013) at 4°C for 15 min. Protein concentrations were determined using the Pierce 660 nm Protein Assay (Thermo Fisher Scientific, Waltham, MA, USA), and equal amounts of total protein (20 µg per lane) were subjected to western blotting. Samples were mixed with NuPAGE LDS Sample Buffer (4×; Cat. No. NP0007, Thermo Fisher Scientific) and NuPAGE Sample Reducing Agent (10×; Cat. No. NP0004, Thermo Fisher Scientific) and heat‐denatured at 70°C for 10 min. Proteins were separated on 10%–20% gradient sodium dodecyl sulfate–polyacrylamide gels at 200 V for 35 min and transferred onto Immobilon‐P polyvinylidene fluoride membranes (MilliporeSigma, Burlington, MA, USA) using a Trans‐Blot Turbo Transfer System (Bio‐Rad Laboratories, Hercules, CA, USA) with the Mixed MW program. The membranes were blocked with 5% skim milk in PBS for 1 h at room temperature and then incubated with the unconjugated primary antibodies described above (1 µg/mL) for 1 h at room temperature. After washing, the membranes were incubated with HRP‐conjugated goat anti‐rabbit IgG (H + L) (1:8,000; Cat. No. 458, MBL Co. Ltd., Nagoya, Japan) for 1 h at room temperature. For the detection of α‐tubulin, the membranes were incubated with HRP‐conjugated anti‐α‐tubulin antibody (1 µg/mL) for 1 h at room temperature, without incubation with a secondary antibody. Immunoreactive bands were visualized using ECL Prime Western Blotting Detection Reagent (Cytiva, Marlborough, MA, USA) and a ChemiDoc Touch MP Imaging System (Bio‐Rad Laboratories). Densitometric analysis was performed using ImageJ (National Institutes of Health, Bethesda, MD, USA).

### 2.5 Enzyme‐Linked Immunosorbent Assay (ELISA)

To evaluate chemokine production at the protein level, IL‐8 and CCL5 concentrations in culture supernatants were measured using a Quantikine Human IL‐8/CXCL8 ELISA Kit (Cat. No. S8000C; R&D Systems, Minneapolis, MN, USA) and a Quantikine Human CCL5/RANTES ELISA Kit (Cat. No. SRN00B; R&D Systems), respectively, according to the manufacturer's instructions. Culture supernatants were collected under the experimental conditions and at the time points indicated in Figure 4B. Each sample was measured in technical triplicate, and the concentrations were calculated from the corresponding standard curve.

### 2.6 Statistical Analysis

Statistical analyses were performed using GraphPad Prism 8 software (GraphPad Software, San Diego, CA, USA). Comparisons between two groups were conducted using the Student's *t*‐test, and comparisons among three or more groups were performed using Dunnett's test or Tukey's test. Statistical significance was defined as *p* < 0.05.

## 3 Results

### 3.1 TLR2 Stimulation Induces Inflammatory Chemokines in PDLFs, Whereas *P. g*. LPS Induces Minimal Responses

The concentrations of Pam3CSK4 and *P. g*. LPS used in this study were determined based on dose–response analyses and confirmed to be non‐cytotoxic by LDH release assays (Abe et al. 2026). Stimulation with Pam3CSK4 markedly induced the expression of *IL8* and *CCL5* at 6 h in PDLFs (Figure 1A). In contrast, *P. g*. LPS did not induce the expression of these chemokines (Figure 1B). A similar pattern was observed after 24 h (Figure 1C).

**Figure 1:**
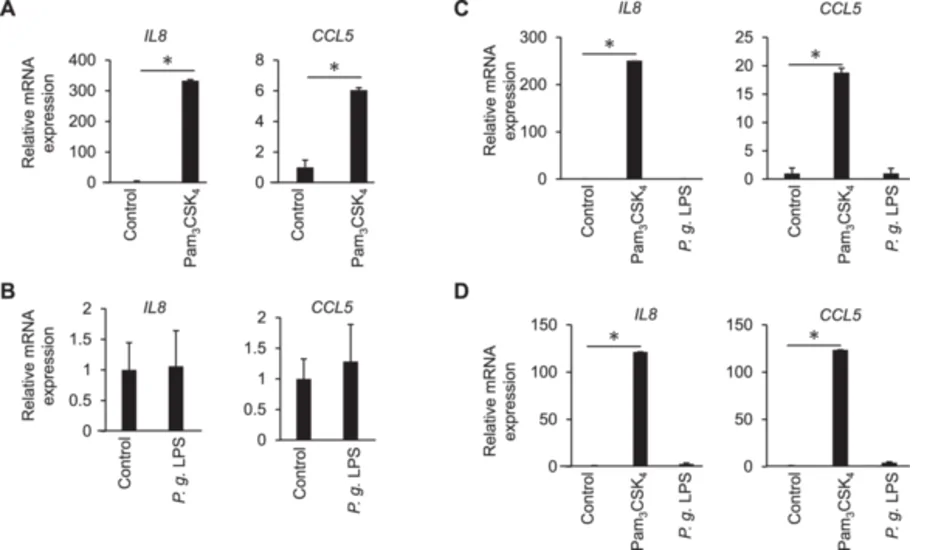
Pam3CSK4 induces inflammatory chemokines in PDLFs, whereas *P. g.* LPS induces minimal responses. (A) PDLFs were stimulated with Pam3CSK4 (TLR2 ligand; 1 µg/mL) for 6 h. (B) PDLFs were stimulated with *P. g*. LPS (TLR4 ligand; 1 µg/mL) for 6 h. (C) PDLFs were stimulated with Pam3CSK4 (1 µg/mL) or *P. g*. LPS (1 µg/mL) for 24 h. (D) To check the reproducibility, PDLFs derived from an independent donor were similarly stimulated with Pam3CSK4 (1 µg/mL) or *P. g*. LPS (1 µg/mL) for 6 h. The indicated mRNA levels were quantified by real‐time RT‐PCR. Data represent mean ± standard deviation from one representative experiment performed in triplicate out of three independent experiments. Statistical analysis was performed using Student's *t*‐test (A, B) or Dunnett's test (C, D). *p < 0.05.

We examined PDLFs derived from independent donors to assess reproducibility across donors. Cytotoxicity of test compounds was assessed by LDH release, as described previously (Inomata et al. 2022). Consistent with the initial findings, Pam3CSK4 induced *IL8* and *CCL5* expression, whereas *P. g*. LPS did not (Figure 1D). Thus, TLR2‐mediated induction of inflammatory chemokines was a robust and reproducible response across two independent batches of PDLFs.

### 3.2 TLR2 Strongly Activates MAPK and NF‐κB Signaling Compared With *P. g*. LPS

We analyzed the activation of MAPK and NF‐κB pathways to investigate differences in downstream signaling induced by Pam3CSK4 and *P. g*. LPS. Pam3CSK4 stimulation induced phosphorylation of p38 and ERK within 15 min and promoted IκB degradation (Figure 2). In contrast, *P. g*. LPS elicited minimal activation of these pathways (Figure 2). Thus, TLR2 stimulation activated major inflammatory signaling cascades in PDLFs while *P. g*. LPS elicited minimal activation.

**Figure 2:**
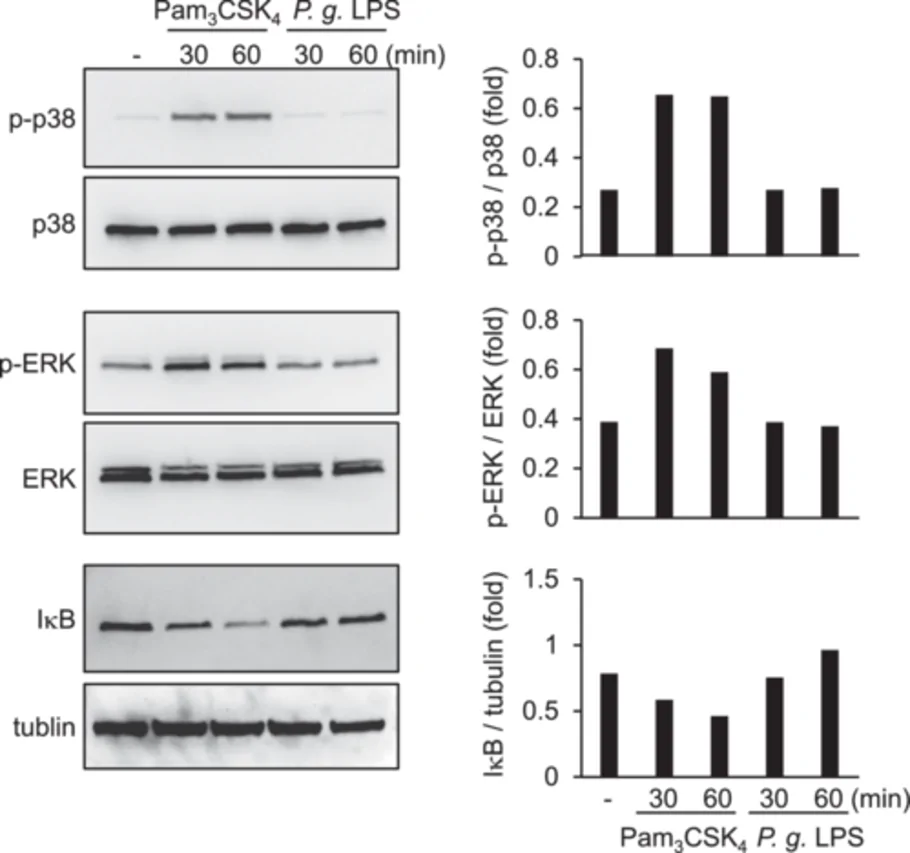
Pam3CSK4 strongly activates MAPK and NF‐κB signaling compared with *P. g.* LPS. PDLFs were stimulated with Pam3CSK4 (1 µg/mL) or *P. g*. LPS (1 µg/mL) for the indicated times. Phosphorylation of p38, ERK, and IRF3, as well as degradation of IκBα, was assessed by western blotting. α‐Tubulin was used as the loading control. Densitometric quantification was performed for all immunoblot bands. Phosphorylated p38 and ERK levels were normalized to total p38 and total ERK, respectively, and IκBα levels were normalized to α‐tubulin. The results shown are representative of three independent experiments.

### 3.3 *P. g*. LPS Induces Weak IFN‐Related and Regulatory Gene Responses in PDLFs

IFNB1 encodes interferon‐β, a type I interferon induced downstream of TRIF–IRF3 signaling and involved in antiviral and inflammatory regulation (Kawasaki and Kawai 2014). We examined the induction of IFN‐related genes to characterize distinct gene expression programs induced by Pam3CSK4 and *P. g*. LPS. Pam3CSK4 did not alter *IFNB1* expression (Figure 3A). In contrast, *P. g*. LPS induced *IFNB1* expression in PDLFs from one donor (Figure 3A), whereas PDLFs from another donor showed minimal induction, suggesting donor‐dependent variability. Under our experimental conditions, IRF3 phosphorylation was not detected by western blotting.

**Figure 3:**
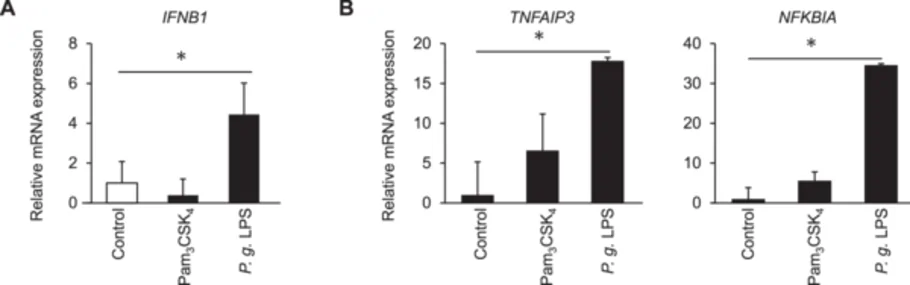
*P. g.* LPS induces weak IFN‐related and regulatory gene responses in PDLFs. PDLFs were left unstimulated or stimulated with Pam3CSK4 (1 µg/mL) or *P. g*. LPS (1 µg/mL). The mRNA expression levels of (A) *IFNB1* and (B) *TNFAIP3* and *NFKBIA* were quantified by real‐time RT‐PCR. Data represent the mean ± standard deviation from one representative experiment performed in triplicate. Statistical analysis was performed using Dunnett's test. *p < 0.05 versus the unstimulated control.

TNFAIP3 is a ubiquitin‐modifying enzyme that terminates NF‐κB signaling, whereas NFKBIA encodes IκBα, a cytoplasmic inhibitor that prevents NF‐κB nuclear translocation (Kawasaki and Kawai 2014). In addition to IFN‐related genes, *P. g*. LPS stimulation induced the expression of the negative‐regulatory genes *TNFAIP3* and *NFKBIA*, whereas Pam3CSK4 stimulation did not significantly increase their expression (Figure 3B). These findings suggest that the IFN‐related and regulatory responses of PDLFs to *P. g*. LPS are relatively weak and may vary among donors.

### 3.4 *P. g.* LPS Pre‐Stimulation Modulates Subsequent TLR2 Responses in PDLFs

To examine TLR crosstalk, PDLFs were first stimulated with *P. g*. LPS for 6 h, followed by Pam3CSK4 stimulation either after washing or without washing. We then analyzed the TLR2 responses under these conditions. When PDLFs were stimulated with *P. g*. LPS for 6 h, washed, and subsequently treated with Pam3CSK4, only *IL8* expression was enhanced (Figure 4A), whereas those of *CCL5*, *IFNB1*, *TNFAIP3*, and *NFKBIA* remained largely unchanged (Figure 4A).

**Figure 4:**
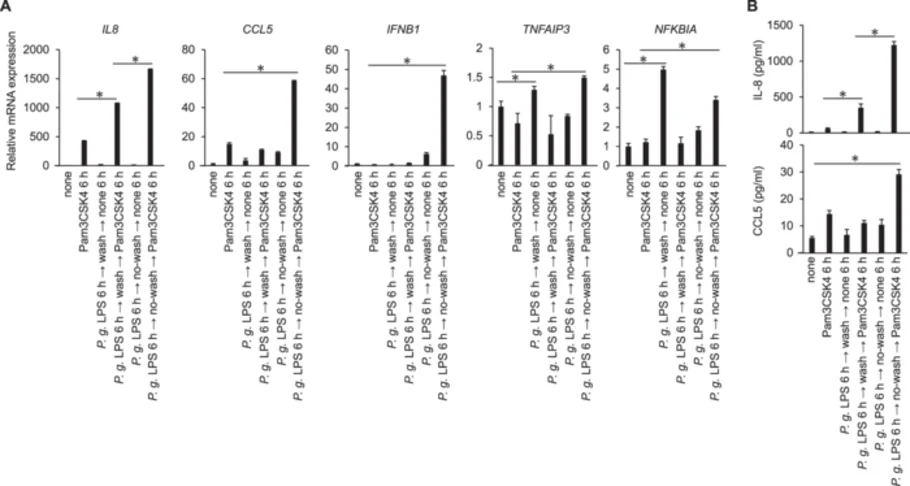
*P. g.* LPS pre‐stimulation modulates subsequent TLR2 responses in PDLFs. PDLFs were pre‐stimulated with *P. g*. LPS (1 µg/mL) for 6 h. The cells were either washed or left unwashed and were subsequently stimulated with Pam3CSK4 (1 µg/mL) for 6 h. (A) The indicated mRNA expression levels were quantified by real‐time RT‐PCR. (B) IL‐8 and CCL5 concentrations in culture supernatants collected under the same experimental conditions were measured by ELISA. Experiments were performed three times independently, and each sample was measured in technical triplicate. Data represent the mean ± standard deviation from one representative experiment. Statistical analyses for both panels were performed using one‐way analysis of variance followed by Tukey's multiple‐comparisons test. *p < 0.05 for the comparisons indicated by the horizontal lines.

In contrast, when Pam3CSK4 was added without washing after *P. g*. LPS stimulation, *IL8*, *CCL5*, *IFNB1*, *TNFAIP3*, and *NFKBIA* expression levels increased (Figure 4A).

Notably, *TNFAIP3* and *NFKBIA* were more strongly induced after 6 h of *P. g*. LPS stimulation, followed by washing, than after continuous *P. g*. LPS stimulation (Figure 4A). These findings indicate that the persistence of *P. g*. LPS stimulation critically influences both TLR2‐mediated chemokine induction and the regulation of negative feedback molecules in PDLFs.

To determine whether the observed changes in chemokine mRNA expression were reflected at the protein level, IL‐8 and CCL5 concentrations in culture supernatants were measured by ELISA. The secretion patterns of both chemokines were generally consistent with their corresponding mRNA expression profiles (Figure 4B). These results provide additional protein‐level evidence supporting the enhancement of chemokine responses under the unwashed condition.

### 3.5 PDLFs Constitutively Express TLR2 and TLR4 Signaling Components

Analysis of receptor expression revealed that PDLFs constitutively expressed *TLR2*, *TLR4*, *LY96*, and *CD14* mRNAs (Figure 5A). Moreover, *TLR2* and *CD14* mRNA expression tended to increase following stimulation with either Pam3CSK4 or *P. g*. LPS (Figure 5A). Western blotting confirmed the expression of TLR2 and TLR4 proteins in unstimulated PDLFs and in PDLFs stimulated with Pam3CSK4 or *P. g*. LPS. TLR2 band intensity appeared higher after Pam3CSK4 and *P. g*. LPS stimulation, whereas TLR4 band intensity remained relatively unchanged (Figure 5B). These findings suggest that changes in TLR2 expression may contribute, at least in part, to the modulation of PDLF sensitivity to bacterial stimuli.

**Figure 5:**
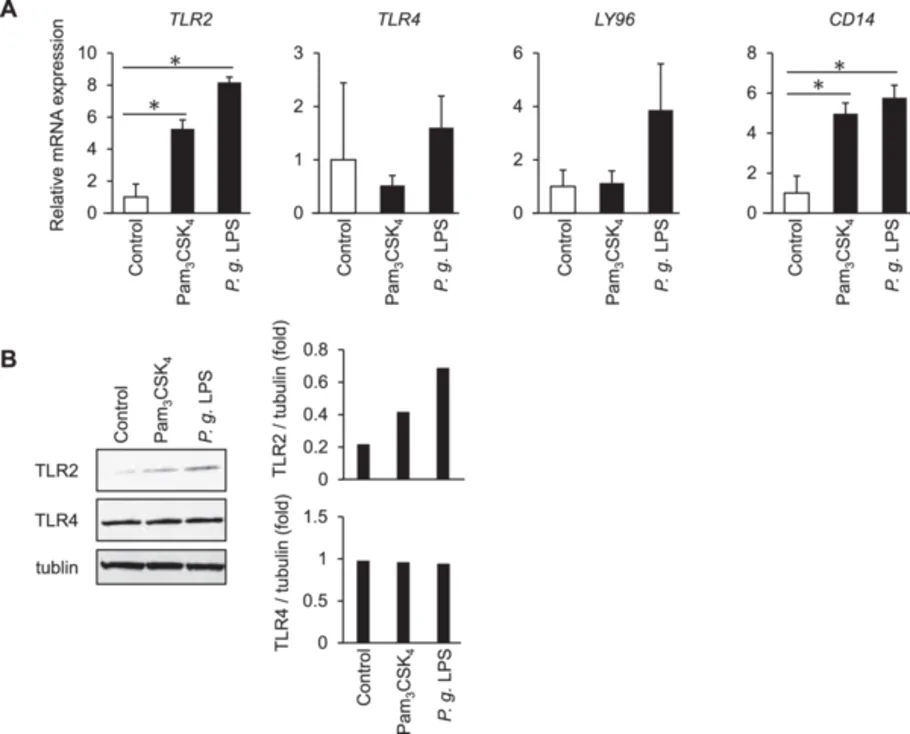
Expression of TLR2 and TLR4 signaling components in PDLFs. (A) PDLFs were left unstimulated or stimulated with Pam3CSK4 (1 µg/mL) or *P. g*. LPS (1 µg/mL), and the indicated mRNA expression levels were quantified by real‐time RT‐PCR. (B) TLR2 and TLR4 protein expression was assessed by western blotting in unstimulated PDLFs and in PDLFs stimulated with Pam3CSK4 (1 µg/mL) or *P. g*. LPS (1 µg/mL) for 6 h. α‐Tubulin was used as the loading control. TLR2 and TLR4 band intensities were normalized to α‐tubulin. The results shown are representative of three independent experiments. *p < 0.05 versus the unstimulated control.

## 4 Discussion

### 4.1 Overview of Findings

This study demonstrates that human PDLFs respond differentially to Pam3CSK4 and ultrapure *P. g*. LPS and integrate these microbial signals in a context‐dependent manner. Pam3CSK4 acts as a strong driver of inflammatory chemokine induction, whereas *P. g*. LPS alone elicits comparatively weak inflammatory signaling but modulates subsequent TLR2‐dependent responses depending on stimulus persistence.

### 4.2 Differential Inflammatory Responses to Pam3CSK4 and *P. g*. LPS in PDLFs

Pam3CSK4 stimulation robustly induced IL8 and CCL5 expression and activated MAPK and NF‐κB pathways, demonstrating a strong pro‐inflammatory phenotype. In contrast, ultrapure *P. g*. LPS failed to induce these chemokines and elicited minimal MAPK or NF‐κB activation under the present experimental conditions. Importantly, these findings should not be interpreted as evidence that TLR4 agonists generally induce weak responses in PDLFs. The biological activity of *P. g*. LPS can differ from that of canonical enterobacterial LPS or synthetic TLR4 agonists, and atypical LPS structures have been reported to produce distinct TLR2/TLR4 interactions and signaling outcomes (Francisco et al. 2021). Previous work in gingival fibroblasts also indicates that responses to TLR2 and TLR4 ligands can differ in magnitude (Uehara and Takada 2007). Thus, the weak direct inflammatory response observed here is most appropriately attributed to the specific *P. g*. LPS preparation and the PDLF experimental context rather than generalized to all TLR4 ligands. Direct comparison with *E. coli* LPS or a synthetic TLR4 agonist will be important in future studies.

### 4.3 Constitutive Expression of TLR Signaling Components and Functional Implications

PDLFs constitutively expressed *TLR2*, *TLR4*, *LY96*, and *CD14* mRNAs. Western blotting further confirmed TLR2 and TLR4 protein expression in unstimulated cells and after 6 h of stimulation with Pam3CSK4 or *P. g*. LPS, with α‐tubulin used as the loading control. These protein‐level data strengthen the evidence that PDLFs possess the receptor machinery required to sense both TLR2 and TLR4 ligands. However, receptor expression alone cannot explain the marked functional differences between Pam3CSK4‐ and *P. g*. LPS‐mediated responses. Distinct downstream pathways, particularly the MyD88‐dependent inflammatory cascade and the TRIF‐dependent interferon pathway, likely contribute to these divergent outcomes (Kawasaki and Kawai 2014).

### 4.4 Weak and Donor‐Dependent IFN Responses to *P. g*. LPS Stimulation

*P. g*. LPS stimulation induced *IFNB1* expression in PDLFs from one donor, whereas the other donor showed minimal induction. No IRF3 phosphorylation was detected in either donor. These findings suggest that interferon responses to *P. g*. LPS in PDLFs are weak and subject to inter‐individual variability. Fibroblasts generally mount attenuated type I interferon responses relative to macrophages or dendritic cells (Ivashkiv and Donlin 2014; Schoggins 2014), supporting the cell‐type‐specific nature of the present results. This attenuated interferon responsiveness may reflect the intrinsic regulatory state of non‐professional immune cells, in which the TRIF–IRF3 signaling axis is often tightly controlled to prevent excessive interferon production in non‐immune tissues (Ivashkiv and Donlin 2014).

### 4.5 *P. g.* LPS Pre‐Stimulation Modulates Subsequent TLR2 Responses

A key finding of this study was that *P. g*. LPS pre‐stimulation modulated subsequent TLR2 responses in a manner dependent on the removal of residual LPS. When cells were washed after *P. g*. LPS stimulation, enhancement of TLR2 responses was limited, and *IL8* was the primary chemokine affected. In contrast, when cells were not washed, *IL8* and *CCL5* expression were markedly amplified, and *IFNB1* expression exceeded that induced by *P. g*. LPS alone. ELISA analyses further showed that the secretion patterns of both IL‐8 and CCL5 were generally consistent with their corresponding mRNA expression profiles (Figure 4B). Persistent exposure to *P. g*. LPS, including residual extracellular LPS under unwashed conditions, may enhance TLR2 signaling. In immune cells, TLR4 stimulation typically induces endotoxin tolerance, characterized by reduced responsiveness to subsequent stimuli (Biswas and Lopez‐Collazo 2009). However, in this study, PDLFs pre‐exposed to *P. g*. LPS exhibited enhanced rather than suppressed TLR2 responses. This divergence highlights cell‐type‐specific mechanisms of TLR signal integration. Consistent with this interpretation, human PDLFs do not develop endotoxin tolerance following *P. g*. LPS stimulation and maintain robust cytokine production upon repeated exposure (Blufstein et al. 2018). Therefore, fibroblasts may contribute to the amplification of chemokine‐mediated leukocyte recruitment rather than tolerance induction, consistent with their emerging role as active regulators of tissue inflammation (Hajishengallis and Lamont 2021; Nilsson 2021). Although cells were thoroughly washed after ultrapure *P. g*. LPS stimulation under the washed condition, the possibility of minimal residual LPS cannot be completely excluded. However, the marked differences between washed and unwashed conditions strongly suggest that the persistence of *P. g*. LPS stimulation primarily accounts for the observed responses.

These observations suggest that prior exposure to *P. g*. LPS may alter the intracellular regulatory environment of PDLFs, thereby modifying the sensitivity of subsequent TLR2 signaling rather than directly inducing inflammatory chemokine expression. These findings are consistent with previous reports demonstrating functional interactions between TLR2 and TLR4, including heterodimer formation under atypical LPS conditions (Francisco et al. 2021). Broader reviews have also highlighted cooperative roles of TLR2 and TLR4 in coordinating physiological and pathological inflammation (Mansouri et al. 2025).

### 4.6 Regulation of Negative‐Feedback Molecules by *P. g*. LPS and Implications for Signal Integration

*TNFAIP3* and *NFKBIA* are central negative regulators of NF‐κB signaling (Kawasaki and Kawai 2014). In the present study, *P. g*. LPS pre‐stimulation modulated the expression of these negative‐regulatory genes in a stimulus‐duration‐dependent manner. Both *TNFAIP3* and *NFKBIA* were more strongly induced under washing conditions than under continuous *P. g*. LPS stimulation, suggesting that transient exposure to *P. g*. LPS preferentially promoted feedback regulation.

In contrast, under unwashed conditions following *P. g*. LPS stimulation, *TNFAIP3* and *NFKBIA* expression was elevated relative to Pam3CSK4 stimulation alone, indicating that persistent *P. g*. LPS stimulation enhanced both inflammatory and regulatory gene expression in combination with TLR2 stimulation. This pattern suggests that PDLFs differentially interpret transient and persistent microbial stimulation, with transient *P. g*. LPS exposure associated with stronger induction of negative‐feedback molecules, whereas prolonged stimulation was associated with combined inflammatory and regulatory gene expression.

Our findings suggest that *P. g*. LPS can modulate inflammatory feedback mechanisms in PDLFs in a context‐dependent manner. This behavior highlights the context‐dependent signal‐integration properties of PDLFs. Mechanistic experiments such as knockdown of *TNFAIP3* or *NFKBIA* were beyond the scope of this study, but future work will be required to determine their causal contributions to TLR signal integration.

### 4.7 Clinical Implications

The present findings may have implications for understanding inflammatory regulation in periodontal tissues, where resident cells are exposed to bacterial components in a dynamic and persistent manner. Our results suggest that the duration and persistence of bacterial stimulation may influence how PDLFs respond to subsequent microbial signals. In particular, persistent exposure to *P. g*. LPS enhanced subsequent TLR2‐dependent chemokine responses, whereas removal of *P. g*. LPS resulted in more limited enhancement.

Although the washed and unwashed conditions used in this in vitro study do not directly reproduce the complex microbial environment of periodontal tissues, they provide a simplified experimental model for examining the effects of transient versus persistent bacterial stimulation. Under the present experimental conditions, *P. g*. LPS did not act primarily as a direct inflammatory stimulus in PDLFs but rather modulated subsequent TLR2‐dependent responses according to stimulus persistence. These findings support the concept that PDLFs may actively integrate sequential or persistent microbial signals and thereby contribute to the regulation of local inflammatory responses in periodontal tissues.

### 4.8 Strengths and Limitations

The strengths of this study include the use of purified TLR ligands (Pam3CSK4 and ultrapure *P. g*. LPS), the integrated assessment of chemokine expression, signaling activation, and receptor expression, and the experimental design incorporating washed and unwashed conditions to evaluate the impact of stimulus persistence on TLR crosstalk.

The limitations include the in vitro nature of the study, the lack of comparison with other cell types, the use of only two independent PDLF preparations, and the absence of direct comparison between *P. g*. LPS and other well‐established TLR4 agonists, such as *E. coli* LPS or synthetic TLR4 agonists. Therefore, the present findings should be interpreted specifically in the context of ultrapure *P. g*. LPS rather than generalized to all TLR4 ligands. Although two independent PDLF preparations were examined and consistently showed Pam3CSK4‐dominant chemokine induction, variability between the preparations, particularly in IFN‐related responses, was observed. Future studies using additional donors and alternative TLR4 agonists will be required to determine the generalizability of these findings.

Additionally, the controlled in vitro conditions using purified ligands may not fully recapitulate the complexity of polymicrobial stimulation in periodontal tissues, underscoring the need for studies incorporating more physiologically relevant microbial environments.

## 5 Conclusions

PDLFs integrate signals from Pam3CSK4 and *P. g*. LPS in a context‐dependent manner. Pam3CSK4 functions as a strong inflammatory stimulus, whereas *P. g*. LPS acts primarily as a priming signal that modulates subsequent TLR2‐dependent chemokine responses according to stimulus persistence. These findings highlight the active role of PDLFs in shaping inflammatory signaling within periodontal tissues. Future studies should directly compare *P. g*. LPS with other TLR4 agonists and determine whether reversing the order of Pam3CSK4 and *P. g*. LPS exposure produces distinct patterns of signal integration or regulatory feedback.

## Author Contributions

Conceptualization: Megumi Inomata and Hiroshi Sakagami. Methodology: Megumi Inomata. Validation: Megumi Inomata. Formal analysis: Megumi Inomata and Masayo Abe. Investigation: Megumi Inomata, Masayo Abe, and Shintaro Oda. Resources: Megumi Inomata and Hiroshi Sakagami. Writing – original draft preparation: Megumi Inomata. Writing – review and editing: Masayo Abe and Hiroshi Sakagami. Visualization: Megumi Inomata, Shintaro Oda, and Hiroshi Sakagami. Supervision: Megumi Inomata and Hiroshi Sakagami. Project administration: Megumi Inomata and Hiroshi Sakagami. Funding acquisition: Megumi Inomata and Masayo Abe.

## Ethics Statement

This study used commercially available primary PDLFs and primary PDLFs isolated from human periodontal ligament tissue. The use of commercially available PDLFs did not require ethical approval. Donor‐derived PDLFs were established from periodontal ligament tissue obtained from a mandibular first premolar extracted from a 12‐year‐old female for orthodontic reasons. The study was conducted in accordance with the guidelines of the Institutional Review Board of the Meikai University Ethics Committee (approval number A0808), and written informed consent was obtained before tissue collection.

## Conflicts of Interest

The authors declare no conflicts of interest.

## Artificial Intelligence (AI) Disclosure

During the preparation of this manuscript, large language model (ChatGPT, OpenAI) was used solely for language editing and stylistic improvements in select paragraphs. No sections involving the generation, analysis, or interpretation of research data were produced by generative AI. All scientific content was created and verified by the authors. Furthermore, no figures or visual data were generated or modified using generative AI or machine learning–based image enhancement tools.

## Data Availability

The data sets generated and analyzed during the current study are available from the corresponding author upon reasonable request.
